# The Potentiating Response to Accentuated Eccentric Loading in Professional Football Players

**DOI:** 10.3390/sports9120160

**Published:** 2021-11-26

**Authors:** Mark Steven Godwin, Tim Fearnett, Mark Ashton Newman

**Affiliations:** Department of Sport and Nutrition, School of Health, Sport and Food, University College Birmingham, Moss House 3 Holland Street, Birmingham B3 1QH, UK; tim@centralstaffscrossfit.com (T.F.); m.newman@ucb.ac.uk (M.A.N.)

**Keywords:** post-activation potentiation, countermovement jump, football, neuromuscular power, accentuated eccentric loading

## Abstract

The purpose of this study was to assess the acute effect of Accentuated Eccentric Loading (AEL) on countermovement jump (CMJ) height, peak power output (PPO) and peak velocity in male professional footballers using loads of 20% or 40% of body mass (AEL20 or AEL40, respectively). Twenty-three male professional football players (age 24 ± 4.5 years, range 18–34 years; body mass 80.21 ± 8.4 kg; height 178.26 ± 7.62 cm) took part in a randomised, cross-over design to test the potentiating responses of two AEL conditions (AEL20 and AEL40) versus a body weight control group (CON). Mean loads for the two conditions were 15.84 ± 1.70 kg (AEL20) and 31.67 ± 3.40 kg (AEL40). There was no significant difference between the three conditions for jump height (*p* = 0.507, η^2^_G_ = 0.022). There were significant differences in peak power between the groups (*p* = 0.001, η^2^_G_ = 0.154). Post hoc analysis with Bonferroni adjustment showed significantly higher peak power for both AEL conditions compared to the control group, but no significant differences between AEL conditions (CON vs. AEL20, *p* = 0.029, 95% CI −1016.735, −41.815, Cohen’s *d* = −0.56; CON vs. AEL40, *p* = 0.001, 95% CI −1244.995, −270.075, Cohen’s *d* = −0.81; AEL20 vs. AEL40, *p* = 0.75, 95% CI −715.720, 259.201, Cohen’s *d* = −0.24). There was no significant difference between the three conditions for peak velocity (*p* = 0.269, η^2^_G_ = 0.046). AEL using either 20% or 40% of body mass may be used to increase peak power in the countermovement jump in well-trained professional football players.

## 1. Introduction

The effective expression of muscular power is widely considered to be a key determinant in athletic performance [1,2,3,4] and is defined as being the amount of work produced per unit of time and the product of force and velocity [5,6,7]. Peak power output (PPO) is suggested to be the most predictive capacity of athletic performance in anaerobic field athletes [8] and is strongly correlated with jump performance (r = 0.66, *p* < 0.0001) [9]. As a result, peak displacement during jumping has been shown to increase alongside PPO over a 10-week period of strength training or power training (0.06 ± 0.04 m and 10.9 ± 3.7 W·kg^−1^ and 0.06 ± 0.04 m and 9.1 ± 2.4 W·kg^−1^, respectively) [10]. Therefore, developing an athlete’s ability to express peak power is a priority for many strength and conditioning coaches, both chronically over a specific training block, and acutely prior to performance.

One suggested method for enhancing an acute performance prior to an activity is that of post-activation potentiation (PAP), a phenomenon whereby an acute enhancement in muscular contraction occurs following a high-intensity conditioning activity [11,12,13,14]. The specific physiological underlying mechanism of PAP is still not well-understood [11,14,15], with suggestions that any acute enhancement in muscular performance is down to the contractile history of the muscle [16] associated with an increase in phosphorylation of myosin heavy light chains increasing cross-bridge attachment rates [17,18]. However, alternative suggestions have been put forward, such as an increase in muscle temperature, a change in muscle pH, and/or a change in muscle blood flow or water content [14]. Despite the lack of clarity regarding the exact mechanism(s) of PAP, it is widely used as part of warm-ups in training and competition. There is a growing argument that the term PAP should only be used when specific physiological outcome measures are assessed, such as muscle temperature and pH, changes in blood flow, neural drive and stiffness Blazevich and Babault [14] Prieske et al. [19]; the majority of the sport and exercise science literature does not assess such variables and instead focuses on performance outcome measures, as such the term post-activation potentiation enhancement (PAPE) has been suggested [14,19]. Subsequently, any attempt to acutely enhance performance with a conditioning activity will be referred to as PAPE.

A variety of PAPE modalities have been implemented and studied across a range of performance outcomes. A systematic review, including 36 studies, from Dobbs et al. [20] reported that performance was not enhanced by the conditioning activity of heavy loads prior to the vertical jump (effect size (ES) = 0.08, 95% CI −0.04 to 0.21). The prescribed loads ranged between 80% to 100% of one repetition maximum (1 RM) for dynamic exercises and up to 150% maximal voluntary isometric contraction (MVIC) for isometric exercises. Conversely, a small overall effect size for jump performance (ES = 0.31) and a moderate effect size for sprinting (0.50) was found by Seitz and Haff [21] pooled from 47 studies in their systematic review. However, it is worth noting that the effect size was greater for stronger athletes than weaker (ES = 0.41 vs. 0.32) and those with more than 2 years resistance training experience (ES = 0.53 vs. 0.44). The effect size for the type of conditioning activity also varied with plyometrics showing the largest effect size compared to high intensity, moderate intensity, and maximal isometric activity (ES = 0.47, 0.41, 019, −0.09, respectively). Studies included in both of these reviews used a range of methods as the conditioning activity, including dynamic resistance exercises, such as squat and deadlift, along with plyometric activity and isometric squats.

During most resistance training, the absolute external load remains constant and is typically prescribed using a percentage of maximal effort. It is established that eccentric muscle action can produce a similar force, with less muscle activation than when shortening or contracting isometrically, and also greater force production with fewer motor units [22]. Therefore, numerous methods of eccentrically overloading muscles are used to take advantage of this, including tempo eccentric training, flywheel inertial training, plyometric training, and accentuated eccentric loading (AEL) [23]. AEL specifically involves a coupling of an eccentric and concentric action with an eccentric load in excess of the concentric portion of the movement, whilst maintaining similar natural mechanics [24]. Methods to achieve this type of training include the use of elastic bands [25], drop jumps [26], weight releasers [27,28], and manually dropped weights at the end of the eccentric phase of a jump [29]. All of these methods share a similar aim, that is, to increase the load during the eccentric phase of the movement immediately prior to the concentric action.

Chronically, Friedmann-Bette [30] found adaptations to both concentric–eccentric training (CON/ECC) and AEL over a 6-week period with an increase in the quadriceps cross-sectional area (5.8 ± 4.3 cm^2^, vs. 8.0 ± 6.5 cm^2^ respectively, *p* < 0.001) and single leg squat 1 RM (11–15 kg, *p* < 0.001) across both groups. However, only the AEL group showed an increase in squat jump height (between 2 and 3 cm, *p* < 0.05), compared with no significant changes in the CON/ECC group. Similarly, significant acute increases in PPO have been found during drop jump exercise performance, with an 11% (*p* < 0.05) increase in the AEL group compared to a control [31]. An AEL of 20% body mass (BM) during five drop jumps was shown to be the most effective load to acutely develop CMJ height compared to an AEL of 10% and BM alone (*p* ≤ 0.05) in strength-trained athletes following a 2-min recovery period [26]. An AEL of either 20% or 30% BM during a drop jump using an elastic device has also been shown to significantly enhance eccentric impulses and RFD in resistance-trained males, although there was no positive change in jump height [32]. A similar study utilising additional downward tensile force via elastic resistance found an AEL of 30% BM to have a positive effect on vertical ground reaction force (6.34%), power output (23.21%), net impulse (16.65%), and jump height (9.52%) (*p* < 0.05) compared to both BM alone and an AEL of 20% BM [25]. Sheppard et al. [29] used a novel method to provide the additional eccentric overload in their study with high-performance volleyball players. A 10 kg weight plate in each hand was used during the eccentric portion of the countermovement jump and released immediately prior to the upwards phase. Results showed a significant increase in performance across all outcome measures other than peak force (jump height; *p* = 0.001, peak power; *p* = 0.036, and peak velocity; *p* = 0.031) with a large magnitude of effect (1.0, 0.83, and 1.03, respectively).

The majority of the literature regarding AEL has utilised recreationally active individuals [25,26,33,34] or strength-trained individuals [26,30,32,35] with one study focusing on sprint-trained athletes [31]. There is a dearth of literature regarding the use of AEL to develop strength and power in team sport athletes. This is somewhat surprising considering the explosive nature of team sports, such as football [36,37]. Faude et al. [38] evaluated the influence of speed and power relating to goal-scoring in the German national football league. They found that in 83% of goals scored, the preceding action was a powerful action by the goal-scorer. For the scorer, the second most common action were jumps (16%), and these were higher than rotations and change-in-direction sprinting (6% each). For the supporting player, jumps were the third most frequent action, behind straight sprinting and rotations. Due to this shortage of the literature regarding the use of AEL as a conditioning activity within professional football, and the previous recommendation that future research should focus on the acute responses to AEL in athletic populations [24], the aim of the present study is to assess the acute effect of AEL using two different loads (20% BM—AEL20 and 40% BM—AEL40) on jump height, PPO, and peak velocity in male professional football players during the countermovement jump.

## 2. Materials and Methods

### 2.1. Participants

Twenty-seven male professional football players (age 24 ± 4.5 years, range 18–34 years; body mass 80.21 ± 8.4 kg; height 178.26 ± 7.62 cm) participated voluntarily in this study. The players played in the third tier of professional football of the English Football League and were free from any injury that may be exacerbated when undertaking eccentrically biased countermovement jumps. Typically, the players undertake 25 h of training each week plus a match during the in-season. All of the players were familiar with jump training and had undertaken AEL training in the preseason. Furthermore, all of the players were considered well-trained as they had taken part in structured strength and conditioning programmes for over 2 years, which included specific lower limb resistance exercises. All participants were verbally informed of the risks and benefits of the study and written consent was obtained prior to any data collection. Participants were instructed to avoid any deliberate training 24 h before each testing session. The study was approved by the institutional review board and conducted in accordance with the Declaration of Helsinki (2013 version).

### 2.2. Study Design

A randomised cross-over design was used to test the acute potentiating responses of two AEL conditions versus a control condition. The study consisted of three experimental sessions conducted over 28 days, with a minimum of 7 days separating each trial, and was conducted during the competition period of a football season (September to November). Prior to any data collection, participants were randomly allocated into one of three groups using an online random integer set generator (random.org). To control for any test-order effects, the order of the trials was also randomised. Countermovement jump height was obtained for each condition along with peak concentric velocity and peak concentric power. The additional load for the AEL conditions was calculated as 20% and 40% of their body mass (AEL20 and AEL40) and applied using dumbbells equating to 10% and 20% of this load in each hand, respectively. The mean loads used were 15.84 ± 1.70 kg (AEL20) and 31.67 ± 3.40 kg (AEL40) for the two intervention conditions. All of the testing took place at the indoor training ground of the professional football club and each session was conducted in the evening following a minimum of 24 h rest, that is, no matches or training sessions.

### 2.3. Procedures

Height and body mass were taken at the first session (seca 213 and seca 813, Hamburg, Germany). All the sessions started with a 10 min warm-up which the participants were familiar with. The protocol consisted of 5 min cycling between 80 W and 100 W (Wattbike Pro, Nottingham, UK) followed by a series of dynamic movements: 30 double leg skips, 10 unilateral hip flexion/extension standing swings, 5× submaximal broad jumps, 5× single leg bounds, and 10× skaters (lateral single leg hops). This was conducted three times with a gradual increase in intensity up to near maximum (90% of perceived effort). After completion of the warm-up, the participants rested for 3 min before conducting the countermovement jumps. Jump height was obtained using a contact mat (Probotics Just Jump, Huntsville, AL, USA) and peak concentric velocity and peak concentric power via a portable accelerometer (Push Band^TM^, Toronto, ON, Canada). Water was available ad libitum throughout the warm-up and jump-testing.

### 2.4. Jump Protocol

To minimise the influence of arm swings, all jumps were performed with the arms fully extended and close to the side of the body. The Push Band^TM^ was secured to the waist of the participant via a belt provided by the manufacturer and placed centrally on the lower back, in line with the iliac crest. Jump height was recorded via the contact mat and determined via the flight time (FT) method, where jump height equals (FT^2^ × *g*)/8. Gravitational acceleration (*g*) equals 9.81 m·s^2^.

Following the warm-up, three maximal countermovement jumps were recorded for each condition (AEL20, AEL40, or CON). The testing was repeated a further two times on separate sessions until each participant completed the three loading conditions. The control condition was used for subsequent reliability analysis. Following each jump, a 2 min rest period was given. The participants were instructed to rapidly descend to a self-selected depth and then jump maximally, keeping their arms by their sides. For the accentuated eccentric loading conditions, the participants rapidly descended to their self-selected depth, released the dumbbells at the lowest point (end of eccentric phase), and then ascended as quickly as possible with a minimal amortisation phase, keeping their arms by their sides. This process was repeated a further two times until three jumps had been completed. The mean value of the three jumps for each condition was used for analysis.

### 2.5. Statistical Analyses

The sample size was calculated using vertical jump data from a previous systematic review [24]. The mean effect size from the review (0.49), α = 0.05 and power (0.8) was used to calculate the effect size of a repeated-measures ANOVA within-factors design (G*Power, version 3.1) [39]. Based on this data, a sample size of 9 was required. Descriptive data were calculated for all anthropometrical measures and expressed as mean ± standard deviation. The repeated-measures ANOVA analyses were performed using JASP software (JASP Version 0.14.1.0 for Macintosh, JASP (Computer Software]). Reliability analysis was performed using IBM SPSS Statistics for Macintosh (IBM Corp, Version 24.0., Armonk, NY, USA) and was determined using a two-way mixed effects model, using the mean results from the control condition for the Just Jump and Push Band^TM^ [40]. The primary outcome measure was vertical jump height. Peak concentric velocity and peak concentric power were secondary outcomes. Jump height, peak concentric velocity, and peak concentric power were analysed using three separate one-way repeated measures analyses of variance. Statistical significance was accepted when *p* ≤ 0.05 and the magnitude of effects for within subjects was reported by generalised eta squared (η^2^_G_) [41]. Where a significant difference was found, a post hoc analysis with Bonferroni correction was made, and Cohen’s *d* was used for effect size comparisons with Cohen’s *d* values of 0.2, 0.5, and 0.8 and above being classified as small, medium, and large effects, respectively [42]. Lastly, box plots were used for visual interpretation of the results and formulated with Tukey whiskers using BoxPlotR. These represent all data points within 1.5 interquartile range (IQR) for the upper quartile and 1.5 IQR for the lower quartile.

## 3. Results

One participant was injured, and three participants did not complete all three sessions, leaving 23 participants for data analysis. Mauchly’s test indicated that the assumption of sphericity had been met for all outcomes (*p* ≥ 0.05) and three separate one-way repeated measures analyses of variance were used for each outcome measure. Values for each outcome measure are presented in Table 1.

### 3.1. Reliability

Reliability of the Just Jump mat and Push Band^TM^ were 0.870 (95% CI 0.763, 0.938) and 0.858 (95% CI 0.717, 0.935), respectively.

### 3.2. Jump Height

**Figure 1:**
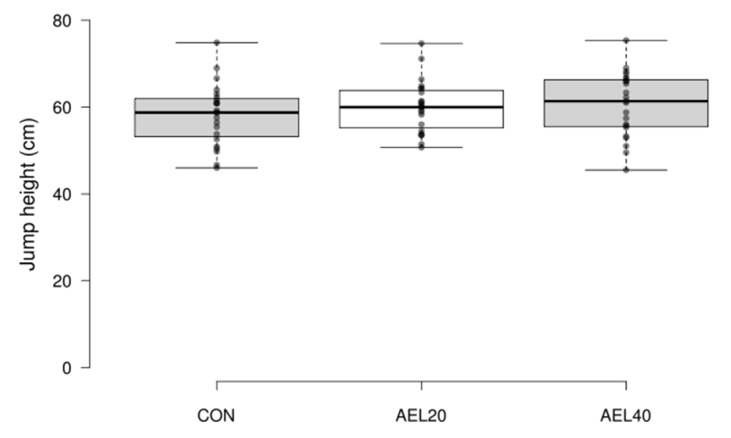
Jump height (cm). No significant differences between groups (*p* = 0.507, η^2^_G_ = 0.022).

There was no significant difference between the three conditions for the primary outcome measure (CON 58.28 ± 7.1 cm, AEL20 60.12 ± 6.0 cm, 60.63 ± 7.4 cm); F (2.44) = 0.690, *p* = 0.507, η^2^_G_ = 0.022) (Figure 1).

### 3.3. Peak Power

**Figure 2:**
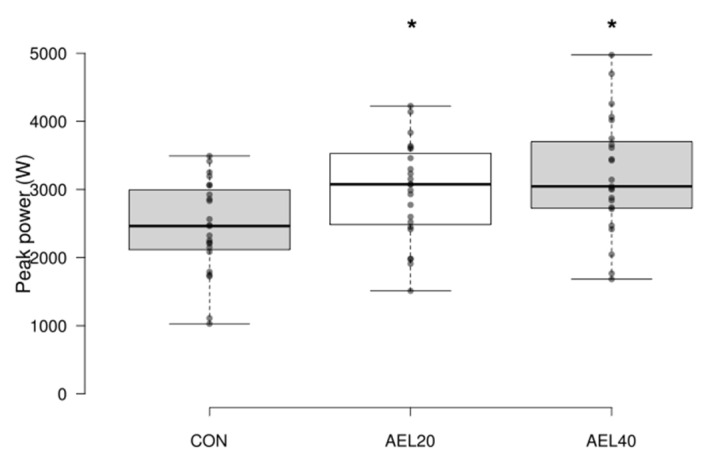
Peak power (W). The asterisk denotes significant differences between both AEL conditions and the control group (*p* = 0.001, η^2^_G_ = 0.154). There was no difference between the AEL conditions (*p* = 0.75).

There were significant differences in peak power between the groups, (CON 2444.9 ± 680.34 W, AEL20 2974.18 ± 725.89 W, AEL40 3202.44 ± 861.99 W; F (2.44), *p* = 0.001, η^2^_G_ = 0.154) (Figure 2). Post hoc analysis with Bonferroni adjustment showed significantly higher peak power for both AEL conditions compared to the control group, but no significant differences between AEL conditions (CON vs. AEL20, *p* = 0.029, 95% CI −1016.735, −41.815, Cohen’s *d* = −0.56; CON vs. AEL40, *p* = 0.001, 95% CI −1244.995, −270.075, Cohen’s *d* = −0.81; AEL20 vs. AEL40, *p* = 0.75, 95% CI −715.720, 259.201, Cohen’s *d* = −0.24). Significant differences were also found for relative peak power (CON 30.5 ± 8 W·kg^−1^, AEL20 37.1 ± 8.3 W·kg^−1^, AEL40 40.3 ± 12.3 W·kg^−1^; F (2.44), *p* = 0.001, η^2^_G_ = 0.157). A significantly higher relative peak power was found for both AEL conditions compared to the control group with no differences between the AEL groups (CON vs. AEL20, *p* = 0.04, 95% CI −12.959, −0.228, Cohen’s *d* = −0.54; CON vs. AEL40, *p* = 0.001, 95% CI −16.208, −3.477, Cohen’s *d* = −0.8).

### 3.4. Peak Velocity

**Figure 3:**
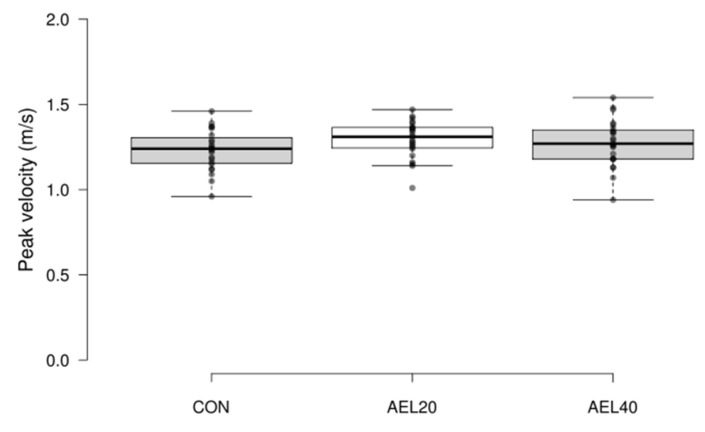
Peak velocity (m·s^−1^). No significant differences between groups (*p* = 0.269, η^2^_G_ = 0.046).

There was no significant difference between the three conditions, (CON 1.23 ± 0.12, AEL20 1.29 ± 0.11 m·s^−1^, AEL40 1.27 ± 0.14 m·s^−1^; F (2.44) = 1.352, *p* = 0.269, η^2^_G_ = 0.046) (Figure 3).

## 4. Discussion

Based on the current lack of evidence, the purpose of the study was to evaluate the acute potentiating effects of AEL on subsequent countermovement jump performance in professional football players. The analysis showed that the increase in load during the eccentric portion of the CMJ failed to elicit any additional stimulus leading to an increase in vertical jump performance. There was, however, significantly greater peak power for both AEL conditions compared to the control group, in line with the findings of Bridgeman et al. [26]. A significant difference when scaled to body mass was also found, with moderate and large effect sizes shown for AEL20 and AEL40, respectively. Despite there being no increase in CMJ height alongside an improvement in peak power, this finding may be of relevance due to the correlation between peak power and a variety of other physical capacities, such as sprint performance [10]. As power is a product of force and velocity, and there was no significant difference in velocity between all conditions, it may be hypothesised, in the absence of a direct force measurement, that there may have been an increase in force production. It has been suggested that an athlete’s strategy for successfully completing ballistic movements is highly individualised, independent of PPO, and instead, dependent on their force-velocity profile [43,44,45], and that individual deficiencies relating to force or velocity values during a ballistic movement may be detected via an individual’s force-velocity profile [46,47]. However, the relevance of individualizing training based upon an athlete’s unique force-velocity profile has recently been questioned [48,49]. After establishing a theoretical optimal squat jump (SJ) force-velocity profile, Lindberg et al. [49] compared the effects of three different training regimes on 10 m and 30 m sprint time, SJ, and CMJ height with participants training either toward or away from their theoretical force-velocity profile, or with a balanced approach to training that did not solely target either of the force-velocity relationship. The authors found there to be no significant group differences for 10 m sprint time (1.0%, −0.9%, and −1.7%), 30 m sprint time (0.9%, −0.6%, and −0.4%), CMJ height (4.3%, 3.1%, and 5.7%), and SJ height (4.8%, 3.7%, and 5.7%) across the ‘training towards’ or ‘training away’ from the optimal profile conditions or following a balanced approach, respectively. As such, future studies relating to the use of AEL conditioning activities to potentiate ballistic movements such as the CMJ should consider the direct measurement of force production along with peak power and velocity measurements to enable further exploration of the force-velocity relationship and the potential causes of an increase in PPO.

Wagle et al. [24] highlighted loading considerations when prescribing AEL. Submaximal loading has been used in a number of studies in an attempt to elicit performance enhancement. Wagle et al. [24] described how this method of AEL could have minimised alteration to the biomechanics of the jump, by allowing a quick transition from eccentric to concentric. If the amortisation phase of the jump was shorter, the participants may have been able to utilise the elastic energy and increase performance [24]. Aboodarda et al. [25] used elastic bands equivalent to 20% or 30% of body mass to provide their accentuated eccentric load prior to a countermovement jump. There was a significant 23% increase in peak power for the 30% AEL condition compared to the control group. Jump height was significantly greater for both AEL groups compared to the control. Peak velocity was only significantly increased in the 30% group. These relatively light loads compared to other studies were able to induce acute performance enhancement for trained male participants, who had undergone plyometric training for a period of 6 months leading up to the trial. Similarly, multiple AEL drop jumps using dumbbells equivalent to 10%, 20%, or 30% were used by strength-trained males (1 RM 1.87 ± 0.27 BM) prior to CMJ performance [26]. CMJ height and peak power was significantly greater for the 20% group (ES = 0.47 and 0.17, respectively). Sheppard et al. [29] used 20 kg of weight plates with high-performance male volleyball players and showed an 11% significant increase in CMJ vertical displacement (ES 1.00), however the same authors also found a significant increase in peak velocity of 16% (ES 1.03) conflicting with the present study. For ballistic type movements, submaximal loading appears to elicit a PAPE response in both acute and chronic intervention studies and therefore could be considered when designing a training programme.

The use of multiple repetitions has been shown as a moderator in submaximal PAPE interventions [50]. In their meta-analysis of PAPE and power, Wilson et al. [51] reported that multiple sets were superior to a single set (ES = 0.24 and 0.66, respectively). They also reinforced the notion that trained participants and athletes achieved significantly greater PAPE effects than untrained, with athletes showing the largest effect size (ES = 0.81 compared to trained, 0.29 and untrained, 0.14). The use of three sets in this study may have contributed to the increase in peak power, but fails to explain the results from jump height or velocity. Therefore, the strength of the athlete plays a role in the prescription of repetitions and should be a factor in training design. Better-trained athletes respond to multiple repetitions, whereas less trained individuals may not.

Two independent meta-analyses [20,21] have shown small effect sizes when using a conditioning activity prior to jump performance (ES 0.31 and 0.08 respectively). However, when the strength of the athlete and training experience were considered, effect sizes increased to 0.41 and 0.53, respectively. In the current study, effect sizes for peak power were 0.56 (AEL20) and 0.24 (AEL40). This similarity for AEL20 may reflect the training status and training experience of the professional football players. Despite the small and moderate effect sizes in the two meta-analyses, a number of moderators have also been proposed, including the type of conditioning activity, number of sets, and rest periods [21]. Traditional high-intensity conditioning activities saw a greater effect size than moderate activities (0.41 vs. 0.19), with plyometric activity showing the largest effect size (0.47). This may support the findings of this study. The proposed mechanisms to explain any increase in performance have been associated with preferential recruitment of type II motor units, higher muscle activation due to an increase in muscle temperature, and an increase in phosphorylation of the myosin light chain [26]. As muscle temperature was not measured in this study, no inference can be drawn on any effect that the warm-up may have had on performance, independent to AEL. However, it is worthy of future investigation as Tsurubami et al. [52] have shown increases in performance following a moderate- or high-intensity warm-up.

An increase in the phosphorylation of the myosin light chain has been suggested as a factor to explain PAPE [14]. Skeletal muscle myosin light chain kinase is found in greater amounts in fast-contracting skeletal muscle compared to slow muscles, and is responsible for the phosphorylation of the regulatory light chain of myosin [18]. Myosin regulatory light chain phosphorylation is induced by maximal or submaximal muscle actions, and has been shown to increase twitch force amplitude, leading to a potentiating effect by altering the myosin motor structure and function. In turn, this enhances the Ca^2+^ sensitivity of the muscle [18]. It is proposed that stronger individuals exhibit a greater percentage of type II muscle fibres, and studies have shown greater responses from well-trained, compared to weaker athletes [21]. Furthermore, under heavier loads, stronger individuals may not get fatigued as rapidly as their weaker counterparts [53]. The participants in this study were familiar with strength-training and had undertaken at least two years of regular conditioning involving compound lower-limb exercises. Despite this, no significant improvement in jump height was observed. Therefore, optimal individualized loading for PAPE may play a role in performance changes, as they may relate to individual strength levels.

## 5. Conclusions

In conclusion, conditioning activities may provide acute performance enhancement and could be used by practitioners; however, moderating factors need to be taken into account. The strength of the athlete, repetitions and rest periods, exercise familiarity, and type of load may play important roles in determining the success of PAPE. AEL is one method that could be used to increase the eccentric phase of a conditioning activity, whilst keeping the overall intensity lower than heavy-resistance exercises. Based on this study, using well-trained professional football players, AEL using either 20% or 40% of body mass may be used to increase peak power in the countermovement jump. However, as no differences were seen between the two AEL conditions, the minimum load to achieve an increase in peak power for this population was 20% of body mass.

## Figures and Tables

**Table 1:** Jump height, peak power, and peak velocity.

Outcome Measure	CON	AEL20	AEL40
Jump height (cm)	58.3 ± 7.1	60.1 ± 6.0	60.6 ± 7.4
Peak power (W)	2444.9 ± 680.3	2974.2 ± 725.9 *	3202.44 ± 861.9 *
Peak power (W·kg^−1^)	30.5 ± 8	37.1 ± 8.3 *	40.3 ± 12.3 *
Peak velocity (m·s^−1^)	1.23 ± 0.1	1.29 ± 0.1	1.27 ± 0.1

Asterisk denotes significant difference between AEL conditions and control group.
